# 

**Published:** 1898-04

**Authors:** 


### CONTENTS.

ORIGINAL ARTICLES: PAGE
Milk-supply from the Bacteriological Standpoint By M. P. RAVENEL, M.D. . . . 215
Why Cows Fail to Breed, and What to do with Them. By GEORGE B. JOBSON, V.S. 221
Etiology of Epizoötic Abortion. By S. J. J. HARGER, V.M.D. . . . . . . 226
Modified Milk for Young Animals. By C. J. MARSHALL, V.M.D. . . . . . 232
What Our Dairy Cattle Inherit. By M. E. CONARD, V.M.D. . . . . . 237
Eversion of the Uterus in Cows. By H. P. KEELEY, V.M.D. . . . . . 239
Pasteurization versus Purity. By H. B. FELTON, V.M.D. . . . . . . 245
Peculiarities of the Lesions of Spavin. By S. J. J. HARGER, V.M.D. . . . . 249
Feeding Animals. By J. CURTIS MICHENER, V.S. . . . . . . . . 253
Tetanus as I Have Found it in Chester County. By W. P. PHIPPS, V.M.D. . . 256
GLEANINGS . . . . . . . . . . . . . . . . . . 259
REPORTS OF CASES:
Clinic Report, Veterinary Hospital, University of Pennsylvania By S. J. J. HARGER, V.M.D. and WM. G. SHAW, V.M.D. . . . . . . . . . . 260
Distressed Breathing in a Cow. By J. F. BUTTERFIELD, V.S. . . . . . . 263
Open Joint. By J. C. MICHENER, V.S. . . . . . . . . . . 264
Is the Actual Cautery Beneficial in Ringbone or Not? By CHARLES BLAND, V.S. 265
Tetanus. By WALKER S. PHILLIPS, V.S. . . . . . . . . . . 266
What Pennsylvanians are Thankful for . . . . . . . . . . . 267
Notes from the University of Pennsylvania Veterinary Department . . . . . 268
EDITORIALS:
The Inauguration of State Editions of the "JOURNAL" . . . . . . . 270
Don't Hide Your Light Under a Bushel . . . . . . . . . . . 270
Ontario's Bad Example Continues . . . . . . . . . . . 271
Pennsylvania's Annual Meeting . . . . . . . . . . . . 273
At It Again . . . . . . . . . . . . . . . . . 273
NECROLOGY . . . . . . . . . . . . . . . . . . 274
SOCIETY PROCEEDINGS:
Pennsylvania State Veterinary Medical Association—U. S. V. M. A.—Pennsylvania Jersey-Breeders' Association—Schuylkill Valley Veterinary Medical Association—Veterinary Medical Association, University of Pennsylvania . . . . . 275
PERSONALS . . . . . . . . . . . . . . . . . . 294

